# Progress in Genito-Urinary Surgery

**Published:** 1901-02-09

**Authors:** 


### Progress in Genito-Urinary Surgery.

**Enlargement of the Prostate.**—Joseph B. Bissel[1] has published a paper on the treatment of this affection. As illustrating the extent to which the cystoscope is now being used, the writer's remark may be quoted that "no surgeon at the present time would open the bladder without first attempting to confirm his diagnosis by the use of the cystoscope." Catheterisation at regular intervals for the removal of residual urine, if available, is spoken of as the treatment of choice, and it may answer for years, the residual urine not increasing, until slowly or suddenly the symptoms become worse from cystitis setting in. Some patients are constantly infecting themselves with catheters, while others escape and seem to be immune to infection. Continuous drainage through a fistula is unsatisfactory, and it is humiliating and depressing to wear a rubber urinal, and infection is common. The radical operations are of two classes: (1) those which aim to remove the obstruction by cutting off the blood and nerve supply, and so to produce atrophy of the gland; and (2) those which affect directly the obstruction itself. Of the first class is the operation of tying the two internal iliac arteries. Bier, of Kiel, and Willy Meyer of New York have tied these vessels, and the latter speaks of the results as being "encouraging in the extreme." Bissel considers the difficulties and dangers of the operation too grave, and the results too uncertain, for him to recommend it. With regard to double castration Bissel says that an analysis of the cases reported shows that in nearly every instance after a time all the symptoms have returned, and he believes that the theory on which the operation is based is as fallacious as the evidences of cure are incorrect. Angeioneurectomy of the cord produces, Bissel says, "just as much atrophy of the prostate as rest in bed, irrigation, and other local treatment would produce, and no more." The operations of ligation, division, or evulsion of the vasa deferentia give similar results, viz. diminution of the size of the prostate for a time, probably due to the reduction of engorgement. The operation of Bottini is next criticised. It is in effect a multiple galvano-caustic division of the middle lobe of the prostate. Willy Meyer's cases, 24 in number, are quoted. He had an 8 per cent. mortality directly due to the operation, and another 8 per cent. mortality indirectly due to it, with 38 per cent. of cures. It presents the dangers of suppression of urine, perforation of the urethra and bladder, and hæmorrhage. Pain and tenesmus and retention of urine and sepsis often follow this operation, and the writer stigmatises the procedure as being unscientific and unsurgical. The theoretical indications for treatment are two: removal of the cause, and removal of the results. The latter can only be approximately effected by constant irrigation of the bladder and its pockets. Bissel then writes favourably of perineal prostatectomy with drainage of the bladder, and the details of that operation are given. A lateral incision is preferred to a mediun one to gain more room. A sound is held in the urethra. The prostate having been reached the tissues are stripped from its apex backwards with a blunt dissector. A vulsellum hooked into the apex of the gland draws it very satisfactorily into view. The capsule "can then be incised on either side of the urethra and each lateral lobe pulled out as a whole, bringing the middle lobe with them." This enucleation is more difficult when the enlargement is rather glandular than fibrous. It is recommended that small portions of prostatic tissue should be left behind rather than risk lacerating the bladder wall. In some cases it is better to incise the membranous urethra and pass the finger into the bladder in order to press down the middle lobe. The bladder is drained by a large tube passed into it through the wound, and is frequently irrigated during the subsequent treatment. Dribbling and incontinence may occur afterwards if the sphincter vesicæ has been injured. J. Shaw M'Claren[2] gives an interesting summary of a paper by Desnos, published in the "Annales des Maladies des Organs Genito-Urinaires," January, 1900, on the Excision of the spermatic veins for enlarged prostate. The operation was simply that for varicocele—excision of the spermatic veins and of a portion of the redundant scrotum. The results of seven cases are reported. In one a man of sixty-two had had varicose veins of the cord and a pendulous scrotum for fifteen years. Frequent and difficult urination had existed for two years, and towards the end there was pus in the urine and residual urine. After the operation urination remained the same for four days, but at the end of a week the frequency had become reduced from fifteen times in the twenty-four hours to six times. Six months later the urine was clear and the prostate much reduced in size. At the end of three years the frequency had rather increased again and the residual urine also, though the latter was less in quantity by half than it had been when the operation was performed. The results at the end of a year in another case, that of a man of sixty-eight, were that the prostate remained small, and the residual urine was fifty grammes. At the time of operation the residual urine had been a hundred grammes. In one of the seven cases the operation was a failure, but the other six benefited. Desnos distinguishes between the varicoceles of young and old men. In the latter the veins are thin and "atrophied." He is inclined to think that the operation leads to "de-congestion" rather than to a real atrophy. F. Leguen (Paris)[3], at the 13th International Congress of Medicine, criticised the remote results of the different operations for enlarged prostate. With regard to castration he said that the results were not so good as early records had made out. There was only amelioration of symtoms, not a permanent cure, and comparing the results of castration with those of other methods, it was doubtful if the advantages were worth the sacrifice. Direct interference with the prostate appeared more and more to be the operation of choice. Patients often relapsed after Bottini's operation. Total prostatectomy could give very good results, and if undertaken by the perineal route before grave complications had set in seemed to be the operation of election for hypertrophy.

**Stricture of the Urethra.**—Reginald Harrison[3] has published a paper on the remote results of structural lesions (*interventions sanglantes*) employed in the treatment of stricture. He remarks that the wound conditions are peculiar, in that they are made in a canal which has to transmit a fluid which under some circumstances is capable of exercising a highly poisonous effect on the tissues and fluids of the body with which it may come in contact. This is shown by the frequency of rigors, and by the occasional suppression of urine, or the occurrence of serious septicæmia. The effects of three kinds of wounds of the urethra are then considered, viz.:—(1) lacerated wounds, such as follow Perrève or Holt's treatment; (2) incised wounds from within, as in Maissoneuve's and other internal urethrotomies; and (3) incised wounds from without, such as Symes. The wounds produced in Holt's method of divulsion of a stricture usually heal not unlike accidental lacerations of the urethra from injury and the liability to the recurrence of stricture is considerable. One form of stricture is mentioned not open to the above objections—viz., the peri-urethral or sub-mucous stricture. In these the dilator ruptures the fibres of the stricture but leaves the mucous lining intact, and the results are in such cases most excellent and are permanent. The proceeding in these cases is comparable to a subcutaneous operation. After internal urethrotomy, in some instances Harrison found on examination after death some years later that no signs of the original stricture were discoverable, showing that permanent cure may result from the operation. But the majority of the cases showed a tendency to relapse within a few weeks or months. In some instances it was apparent that the stricture had not been completely divided, and if in such cases bougies were passed lacerations ensued, and the results were an aggravation of the stricture. After Symes' method Harrison in several instances found no recurrence after an interval of years. Probably the drainage contributed to the good results. Thus, as a whole, Harrison shows that most strictures treated in the three ways referred to showed a tendency to re-contraction. In some this could be easily counteracted by the use of bougies, but in others repeated operations were required, or even the formation of a urinary fistula in place of the urethra. Harrison then alludes to the great value in the prevention of strictures of making a perineal incision for drainage in cases of laceration of the urethra from traumatism. In some instances this treatment has led to healing of the rupture in the urethra just as satisfactorily as after a median cystotomy, and no stricture has followed. In 1885 Harrison published observations on cases in which he had combined the principles of internal and external urethrotomy. The method had, on the whole, given good results, the external urethrotomy being made on the same principles as in the traumatic lacerations alluded to above.

[1] Med. Rec., Nov. 1900. [2] Scottish Med. and Surg. Jour., vol. vii., No. 3. [3] Lancet, Aug. 1900.

